# Supplementary material for: Redox Reactivity of Nonsymbiotic Phytoglobins towards Nitrite
Source: Molecules. 2024 Mar 7;29(6):1200. doi: 10.3390/molecules29061200 (PMC10975543; doi:10.3390/molecules29061200)
Supplement: Supplementary file 1 [file molecules-29-01200-s001.zip › molecules-2757322-supplementary.pdf]

Supplementary material

Redox reactivity of nonsymbiotic phytoglobins towards nitrite

Cezara Zagrean-Tuza [1], Galaba Pato [1], Grigore Damian [2], Radu Silaghi-Dumitrescu [1], Augustin C. Mot [1*]

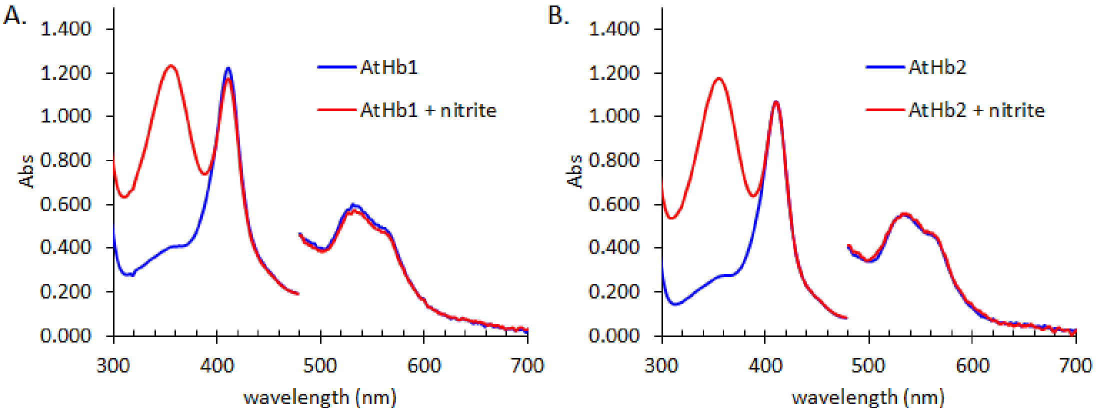

Figure S1. UV-vis spectral changes for the recombinant methemoglobin ($HbFe^{3+}$) of AtHB1 (A0 and AtHb2 (B) with nitrite in aerobic conditions. UV-vis spectra of 8 µM methemoglobin ($HbFe^{3+}$) after addition of nitrite at 40 mM, in 50 mM phosphate buffer pH 7 and 25 °C.

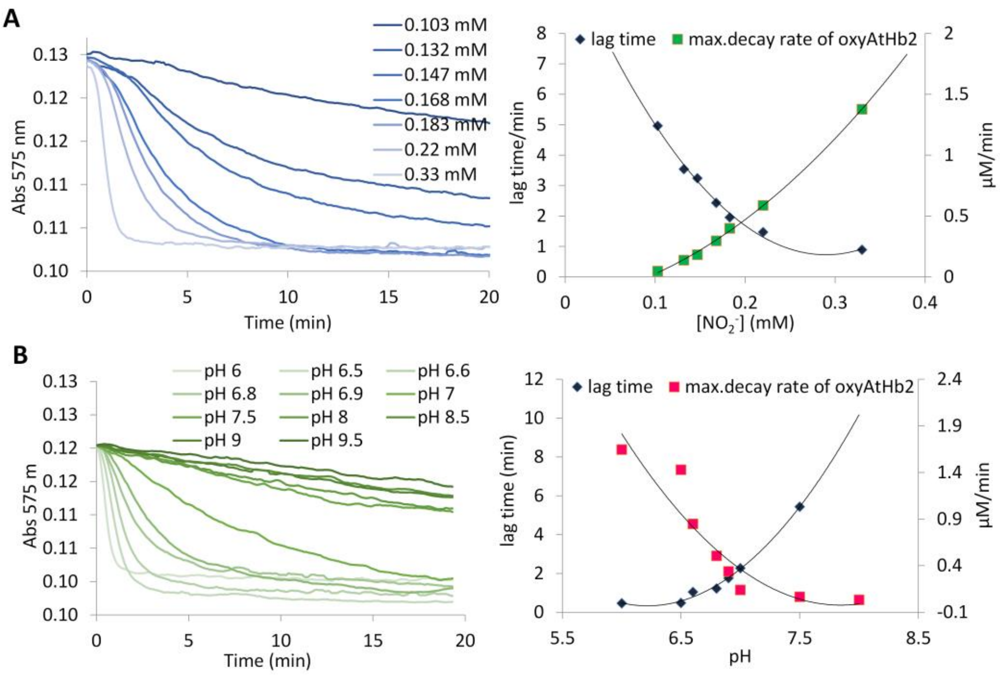

Figure S2. Kinetic profiles and their corresponding lag times and maximum decay rates for the reaction between oxyAtHb2 and nitrite (A) at different concentrations of sodium nitrite, 8µM oxyAtHb2 was incubated with $NaNO_2$ (0.103 - 0.22 mM) in 50 mM phosphate buffer pH 7 **(B)** at different pH values, 8µM oxyAtHb2 was incubated with 0.138 mM $NaNO_2$ in pH 6-9.5.

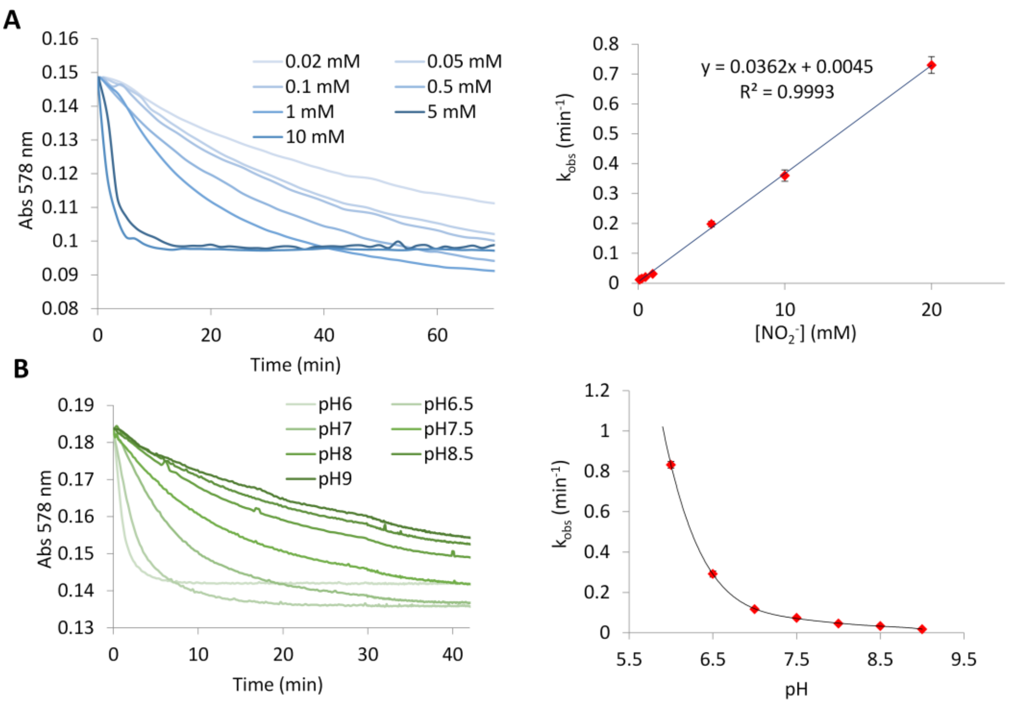

Figure S3. Kinetic profiles and their corresponding k_{obs} values for the reaction between oxyAtHb3 and nitrite (A) at different concentrations of sodium nitrite, 8µM oxyAtHb3 was incubated with $NaNO_2$ (0.02-10 mM) in 50 mM phosphate buffer pH 7 **(B)** at different pH values, 8µM oxyAtHb3 was incubated with 5 mM $NaNO_2$ in pH 6-9.

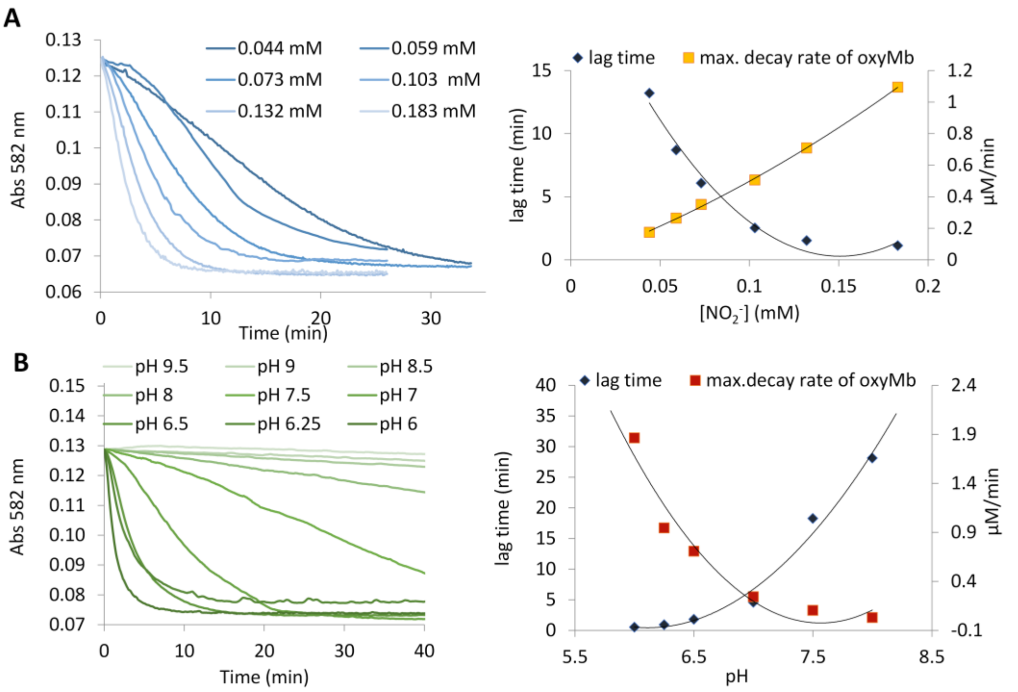

Figure S4. Kinetic profiles and their corresponding lag times and maximum decay rates for the reaction between oxyMb and nitrite (A) at different concentrations of sodium nitrite, 8µM oxyMb was incubated with $NaNO_2$ (0.044-0.183 mM) in 50 mM phosphate buffer pH 7 **(B)** at different pH values, 8µM oxyMb was incubated with 0.073 mM $NaNO_2$ in pH 6-9.5.

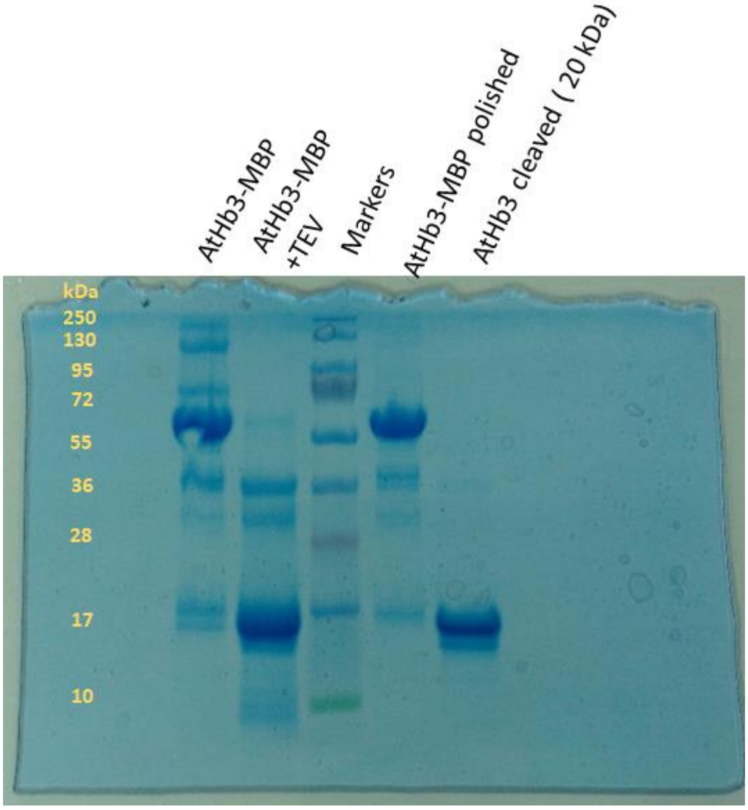

Figure S5. Preparation of pure recombinant AtHb3 phytoglobin. SDS-PAGE showing the TEV mediated His8-MBP removal and purification level of AtHb3. For AtHb1 and AtHb2 see reference: Mot, A.C.; Puscas, C.; Miclea, P.; Naumova-Letia, G.; Dorneanu, S.; Podar, D.; Dissmeyer, N.; Silaghi-Dumitrescu, R. Redox Control and Autoxidation of Class 1, 2 and 3 Phytoglobins from Arabidopsis Thaliana. Sci. Rep. 2018, 8, 13714, doi:10.1038/s41598-018-31922-4.
